# ‘Teach me how to look after myself’: What people with bronchiectasis want from education in a pulmonary rehabilitation setting

**DOI:** 10.1111/crj.13563

**Published:** 2022-11-20

**Authors:** Annemarie L. Lee, Rebecca Smith, Lucy Burr, Anne B. Chang, Chien‐Li Holmes‐Liew, Paul King, Peter Middleton, Lucy Morgan, Daniel Smith, Rachel Thomson, Grant Waterer, Conroy Wong, Rachael McAleer

**Affiliations:** ^1^ Department of Physiotherapy Monash University Frankston Victoria Australia; ^2^ Monash Lung and Sleep Monash Health Clayton Victoria Australia; ^3^ Institute for Breathing and Sleep Austin Health Heidelberg Victoria Australia; ^4^ Department of Allied Health Research Cabrini Health Malvern Victoria Australia; ^5^ Physiotheraphy Department Launceston General Hospital Launceston Tasmania Australia; ^6^ Australian Bronchiectasis Registry Lung Foundation Australia Milton Queensland Australia; ^7^ Department of Respiratory and Sleep Medicine Mater Hospital Brisbane Brisbane Queensland Australia; ^8^ University of Queensland Brisbane Queensland Australia; ^9^ NHMRC Centre for Research Excellence in Paediatric Bronchiectasis (AusBREATHE), Australian Centre for Health Services Innovation, Queensland University of Technology, and Menzies School of Health Research Charles Darwin University Darwin Northern Territory Australia; ^10^ Department of Respiratory Medicine Concord Repatriation General Hospital Concord New South Wales Australia; ^11^ Sydney School of Medicine The University of Sydney Camperdown New South Wales Australia; ^12^ Gallipoli Medical Research Institute Greenslopes Private Hospital Greenslopes Queensland Australia; ^13^ Middlemore Hospital University of Auckland Auckland New Zealand; ^14^ La Trobe Rural Health School La Trobe University Bendigo Victoria Australia

**Keywords:** bronchiectasis, education, pulmonary rehabilitation, self‐management

## Abstract

**Introduction:**

Pulmonary rehabilitation is recommended for people with bronchiectasis. Various education topics are included in these programmes, but the content is largely guided by the needs of people with other respiratory conditions.

**Objectives:**

With the education topics applicable to people with bronchiectasis unclear, we aimed to explore the perspective of adults with this condition on relevant educational topics in a pulmonary rehabilitation context.

**Methods:**

Participants from the Australian Bronchiectasis Registry were invited to undertake a semi‐structured interview. Interview transcripts were coded independently, with themes established by consensus (two researchers).

**Results:**

Twenty‐one people participated. The major themes were greater clarity on the underlying cause of bronchiectasis and prognosis. Most sought knowledge about self‐management strategies and treatments to address extra‐pulmonary symptoms. Participants requested more information on physiotherapy options and the role of exercise and physical activity outside of pulmonary rehabilitation. Preferences were mixed for the education delivery model.

**Conclusions:**

We have identified unmet educational topics of interest for people with bronchiectasis. Our study provides a framework for education topics desired by adults with bronchiectasis within a pulmonary rehabilitation setting. The topics identified will guide development of an education curriculum for pulmonary rehabilitation that is more fit‐for‐purpose for people with bronchiectasis.

## Graphical abstract

In the education component of pulmonary rehabilitation, people with bronchiectasis seek to obtain greater clarity on the underlying cause of their condition and its prognosis. Further knowledge about self‐management strategies, treatment for extra‐pulmonary symptoms and the role of exercise outside of pulmonary rehabilitation as part of management were also desired.

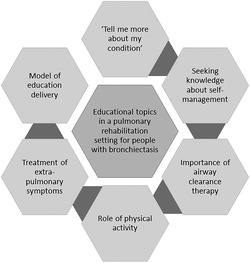

## 1 INTRODUCTION

People with bronchiectasis frequently experience chronic cough and sputum production, have reduced exercise tolerance and poorer quality of life.^1^, ^2^ Treatment options recommended by national and international guidelines include physiotherapy in the form of airway clearance therapy and pulmonary rehabilitation.^1^, ^2^, ^3^ Airway clearance therapy is recommended to be undertaken regularly,^1^, ^2^ while pulmonary rehabilitation should be offered to individuals experiencing limitations in exercise tolerance.^4^

Pulmonary rehabilitation programmes have many components including exercise training, education and behavioural change.^5^, ^6^ Exercise training for those with bronchiectasis is individually prescribed, aligning with the current guidelines for all participants in this intervention,^5^ and usually undertaken for a duration of six to 8 weeks.^7^ It is generally recommended that initiation or optimisation of airway clearance therapy, depending on what is relevant for an individual participant is included within pulmonary rehabilitation.^8^ For the education and behavioural change components, a range of structures have been applied, including didactic lectures, peer‐to‐peer discussion, hands on demonstration and case‐based learning, with no specific recommendations for those with bronchiectasis.^9^ The educational components of pulmonary rehabilitation were originally designed for people with chronic obstructive pulmonary disease (COPD) and may not include topics that are relevant to people with bronchiectasis.^10^ The symptoms, medical and physiotherapy treatments, disease progression and outcomes differ in bronchiectasis from those of COPD. Despite recommendations for education material,^2^ a workshop review of education delivered within pulmonary rehabilitation highlighted the lack of topics that are specifically tailored to all patients' needs.^11^ While patients have a desire to learn more information about their condition, there remains a lack of information material openly accessible for individuals with bronchiectasis.^12^ A lack of knowledge may reduce patient confidence^13^ and is a recognised barrier to treatment adherence.^14^ With poor treatment adherence negatively influencing exacerbations of bronchiectasis,^15^ tailoring educational content to patients' needs is an important step towards facilitating self‐care and supporting individuals to be an active participant in their healthcare.

Proposed educational topics for those with bronchiectasis include flags to identify an acute exacerbation, the role of medications, options for exercise and airway clearance therapy, management of mood disorders, nutrition and vaccinations.^12^ However, the source of these suggestions is unclear and it is unknown whether these fulfil the educational needs from the perspective of people with bronchiectasis. A key priority in the goal of educating people with bronchiectasis is to provide a curriculum which contains desired detail that users want rather than information that healthcare providers decide patients should have. In order to create these resources, the first stage is to identify what educational topics are desired by individuals with bronchiectasis when attending a pulmonary rehabilitation programme.

In the absence of current patients' perspective, our study aimed to explore educational topics of interest which could be a focus in pulmonary rehabilitation by people with bronchiectasis. This is the first step in the development of a suite of educational materials which are suitable for delivery within pulmonary rehabilitation programmes for adults with bronchiectasis.

## 2 METHODS AND METHODS

### 2.1 Participants

A qualitative study design was employed. Participants from the Australian Bronchiectasis Registry were invited to participate through a study advertisement. Individuals were excluded if they were unable to communicate verbally due to language skills, hearing or cognitive impairment. This study has been approved by the institutional review boards at Monash University (approval HREC 18416). All participating patients are requested to provide written informed consent. A total of 63 participants were initially approached, with 41 not responding to the invitation. Recruitment occurred between November 2018 and April 2019.

Recruitment was considered to be completed when data saturation was reached. This occurred following 15 interviews with participants and was further confirmed by an additional six interviews. Demographic information related to age, gender, severity of lung disease (according to spirometry and Bronchiectasis Severity Index^16^ measure) and previous exposure or participation in pulmonary rehabilitation of participants were recorded.

### 2.2 Procedure

Interviews were undertaken using a semi‐structured interview schedule and via telephone by a physiotherapist with experience in managing patients with bronchiectasis (ALL). Average interview duration was approximately 30 min. The content of the interview schedule was informed by a previous study of educational topics of people with respiratory disease^17^ and the findings of a recent systematic review.^10^ Questions covered topics related to the educational needs of people with bronchiectasis. An outline of the interview questions is included in Appendix A.

### 2.3 Analysis

All interviews were audio recorded, de‐identified and transcribed verbatim by a professional transcriber. The data were stored and organised using the computer software program (QSR NVivo Version 12.0; QSR International, Doncaster, Australia). Descriptive and reflective notes were maintained by the interviewer. Thematic analysis was undertaken using grounded theory.^18^ The interview transcripts were independently examined by two investigators (ALL and RS). Transcripts were read line‐by‐line and divided into descriptive codes to represent the data (open coding). Codes were then organised hierarchically to form themes and the original transcripts searched to refine the relationship between themes and codes (axial coding). The final themes were agreed through iterative discussion between the two investigators. Quotations were extracted from the transcripts to support the data for each theme. Data analysis commenced during the period of data collection, in order for preliminary analysis to inform the ongoing interview process.

## 3 RESULTS

### 3.1 Demographics

A total of 21 participants were interviewed. Their demographic details are outlined in Table 1. The majority of participants had severe bronchiectasis according to the Bronchiectasis Severity Index, with a range of aetiologies, similar to previous reports of those with bronchiectasis engaged in pulmonary rehabilitation.^19^, ^20^, ^21^ The degree of exertional dyspnoea ranged from a modified Medical Research Council dyspnoea score of 0 to 2 and a mix of comorbidities were present for participants. Six participants had previously undertaken pulmonary rehabilitation. Six themes were identified.

**TABLE 1:** Participant demographics

Participant ID	Age (years)	Gender (M/F)	Underlying aetiology	FEV_1_ % pred	FVC % pred	BSI	Comorbidities	mMRC dyspnoea score	Previous PR
1	41	M	CTD	76.8	86.1	Mild	NR	0	Yes
2	67	M	Idiopathic	62.8	89.3	Moderate	Asthma	1	Yes
3	85	F	Postinfective	89.2	112.8	Moderate	Anxiety	1	Yes
4	52	F	Idiopathic	72.0	97.4	Severe	Anxiety	1	No
5	79	M	Postinfective	128.0	132.9	Moderate	Depression	1	No
6	79	F	PCD	76.8	90.3	Severe	Rhinosinusitis	2	Yes
7	83	F	Postinfective	N/A	N/A	Severe	NR	2	No
8	69	F	Other	N/A	N/A	N/A	GORD	1	Yes
9	62	F	NTM	101.7	113.6	Severe	Depression	1	No
10	78	F	Postinfective	45.1	57.0	Severe	COPD	1	No
11	65	F	Idiopathic	N/A	N/A	Severe	NR	2	Yes
12	82	F	Idiopathic	69.7	89.2	Severe	Asthma	1	No
13	40	M	Immunodeficiency	78.9	93.4	Moderate	Rhinosinusitis	0	No
14	69	F	Post‐TB	119.7	128.5	Mild	NR	0	No
15	75	F	Idiopathic	56.2	76.4	Severe	COPD	1	No
16	73	F	Postinfective	81.1	93.7	Severe	Depression	1	No
17	70	M	Postinfective	111.2	124.2	Moderate	NR	0	No
18	86	F	Idiopathic	97.6	126.3	Moderate	GORD	1	No
19	77	F	Idiopathic	100.1	114.2	Moderate	NR	0	No
20	72	M	Idiopathic	41.7	79.6	Severe	COPD	1	No
21	46	M	Postinfective	86.0	93.0	Moderate	Asthma	1	No

Abbreviations: BSI, Bronchiectasis Severity Index; COPD, chronic obstructive pulmonary disease; CTD, connective tissue disease; F, female; FEV_1_, forced expiratory volume in 1 s; FVC, forced vital capacity; GORD, gastro‐oesophageal reflux disease; IQR, interquartile range; M, male; mMRC, modified Medical Research Council dyspnoea scale; NA, not available; NR, nothing reported; NTM, nontuberculous mycobacteria; PCD, primary ciliary dyskinesia; PR, pulmonary rehabilitation; TB, tuberculosis.

### 3.2 Themes

#### 3.2.1 Tell me more about my condition

Most participants felt they were aware of the origin of their condition, but some felt further clarity on the aetiology would be beneficial.

It was sort of explained at the time and initially the doctor thought it might have come from the measles … (P08)

… and at that stage they did say that it was probably because of untreated bronchitis as a child. (P14)

but why I actually have it and how I got it, yeah, don't understand all that; where it comes from. (P02)

With regard to understanding their condition from the context of underlying respiratory changes and reasons for symptoms, some participants felt they had received sufficient information while others felt they have not been provided with enough facts.

Really, there's not much more they can tell me really, I do not think apart from what to do to relieve the condition. (P02)

All my life I've found that bronchiectasis sort of just slipped through the cracks, it just doesn't rate … pretty much most of what I've found out through my life is on my own. (P03)

Despite, this, the majority of participants understood their condition was not curable, but that prognosis could be optimised with treatment. However, more information as to what they could expect in the future was desirable by some participants.

This is a disease, it's incurable and you're not going to get better, but you can slow down the process and I think that's important. (P06)

Good treatment can prolong my life and to, ah, make the management of my, ah, disease a little bit easier. (P04)

I do feel there's a gap. I feel I'd like to know a lot more about it and where it's likely to lead. Um I would like to know what sort of—I can only expect it'll get worse. (P16)

#### 3.2.2 Seeking knowledge about self‐management

Most participants recognised the importance of being empowered for self‐management, assuming responsibility, despite the challenges associated with it. Related to this, most participants were interested in learning more information about self‐management strategies that they may be able to apply, including early recognition of an exacerbation.

I've learnt that I've—I've got to be the one to take action. I was determined to help myself. (P04)

Teach me how to look after myself, … of course I'm interested in anything that keeps me well. (P09)

Another really important thing I think is having some understanding of what happens when you get unwell … And what to do, when it's time to you know go to the doctor and when it's time to go to emergency. (P10)

To further facilitate self‐management, some participants desired more information related to the role of diet and lifestyle including sleep.

I didn't get any information about diet and I didn't get any information about um improving my sleeping. I guess strategies for sleeping and resting would be good. (P05)

#### 3.2.3 The importance of airway clearance therapy

The overwhelming majority of participants reported a good understanding of the role of airway clearance therapy for managing chronic cough and sputum production, although some felt their knowledge was impeded by the duration of their diagnosis and information available at that time.

Well, that you've got to keep up your physio. That's the most important thing, because otherwise if you don't, all that phlegm just sits at the bottom of your lungs and then just gets infected and then you're crook. That's about the most beneficial thing I can say that helps me. (P09)

I think my understanding reasonably good um now but it's taken several years to work out how I can manage it because the original diagnosis was done a long, long time ago and I don't think I got very good um information at the time on how to manage it. (P05)

Further information was desired regarding alternative options for airway clearance therapy or guidance as to the frequency of review of their current regimen and advice about cleaning devices.

You know most people didn't really realise the equipment you can use and how to look for it. I had no idea how to clean them or that you had to clean them or how often. (P10)

Well, I think the main thing is to know how to do your drainage properly, um being taught properly and um just having someone to maybe check it that you—because you do, over time, get lazy and forget what you're doing and you don't do it properly. (P02)

#### 3.2.4 The role of physical activity

While some participants expressed that exercise was part of their management, their understanding of the role and benefit was vague. Others stated it was not a major component of their treatment routine, but there was a desire for further information regarding exercise and physical activity, particularly outside of a pulmonary rehabilitation programme and within the home environment.

I probably would like to learn if there's any exercise, you know, like, anything that I can do to improve it. (P11)

I want to know how to be a bit more agile. I've always been very agile, but I'm finding I'm—I'm slowing down a bit and I'm limiting to what I can do. (P17)

It's learning how to do it at home as well because it's not good just going to a program without keeping it up at home … (P11)

#### 3.2.5 Treatment for extra‐pulmonary symptoms

A large number of participants expressed the need for clinicians to provide education regarding the management of extra‐pulmonary symptoms related to bronchiectasis or co‐existing conditions.

You're not just a pair of lungs. You've got other parts of your body, that do breakdown and you don't want that to happen because you've got enough to deal with your lungs. I don't know what I can do to help that. (P07)

Participants highlighted the psychological burden of the condition and a desire for strategies to manage the psychological challenges, including depression and isolation, the invisibility of the condition and the stigma associated with some symptoms.

I mean you can get really down and isolated if you don't have strategies to deal with that. And I think that's a really important thing that I'd pass onto anyone that's new. (P10)

You'd be very sick while you're working and nobody would believe how sick you really were. It's that invisibility of it. (P19)

I bring up phlegm and, you know, that's the most embarrassing thing about lung condition, I reckon. Because people go, ‘You shouldn't even be out with a chest like that’. (P09)

#### 3.2.6 Model of education delivery

Only a small proportion of participants had previously engaged with pulmonary rehabilitation and at least half of participants were unfamiliar with this form of management. Some participants favoured the provision of education in a group setting, including people with a mix of respiratory conditions. Others had concerns that a mixed diagnostic group may detract from the relevance of discussion topics.

I need to mix more with people with the same conditions I think and we learn off of each other too. (P13)

If you've got a group of say five people or six, and if each person has got something different, they each want to know about their thing. You'd rather it be with people who are experiencing the same sort of condition or the same symptoms, just so you can get a little bit more information. (P16)

Other participants were indifferent to group or individual education sessions while a small number of participants had a defined preference for individual education, expressing the desire to maintain a level of anonymity regarding their condition.

Oh I'm indifferent on that. I'd say I'm neither here nor there on that. (P12)

I'm probably a bit of a shy person, where sometimes they might think that my question is not as important to someone else or as significant as something, that someone else has said. (P08)

For those preferring group‐based education, the benefits expressed towards this format included reduced isolation, gaining support, having the opportunity to further their knowledge and relating to individuals with a similar condition.

Sharing, listening and understanding make you feel better and then you're more open to what might be suggested then. (P03)

If you've got a class, it's a support group and—and especially for people that probably wouldn't choose to go to a support group. (P10)

I'm always happy to learn more from them, or listen to them. I've never asked for any advice because I listen to what they say, you know. That's why I find it difficult to know what to say to them or ask them. (P13)

With regard to who should be delivering education sessions, experience in the field was considered the most important factor by participants.

It is very good to have a person of you know at the highest level like the lung specialist, but I think, even the trainees, cause they're up to date with all the latest. (P06)

People like a physiotherapist or an OT or Exercise Physiologist, professionals who have worked for quite some time in those fields. (P08)

Social workers can help with the living with it. Psychologists are very good. The coping of it with respect to technical needs, the physio could cover. (P21)

With regard to retaining information from education sessions, there was a mixed perspective. Some participants prefer notes to refer to, to reinforce key concepts. While some are self‐taught via the internet, others have no desire to use this as a resource.

It's great to have notes to refer back to, whether it has diagrams explaining things to do, things that you've been taught. (P08)

But it's no use telling me to read it on the internet, because I'm, I'm a hard copy person. And the internet gives me the horrors. (P17)

The importance of using layman's terms to provide the information and ensuring a patient's understanding was highlighted by some participants as a practice point and an approach towards educational delivery.

Speak to them in layman's terms. Don't assume that they know certain things. (P16)

Speak in simple language, not the medical way. (P04)

Just really breaking it down and understanding, that's very important. (P10)

## 4 DISCUSSION

This is the first study exploring the perspectives of patients regarding the role, content and model of education within pulmonary rehabilitation for people with bronchiectasis. The results highlight the desire for further information linked to options for self‐management and greater recognition and treatment suggestions of extra‐pulmonary symptoms and the psychological burden of the disease. Educational needs related to advances and alternative airway clearance therapy and the role of physical activity were also highlighted. There were mixed views related to the model of delivering education in this setting.

People with bronchiectasis have a desire for further information regarding the origin of their condition and its prognosis. This is similarly reflected by the United States and European Multicentre Bronchiectasis Audit and Clinical Research Collaboration patient‐centred research priorities of gaining a greater understanding of the cause and natural history of bronchiectasis and the impact of underlying conditions.^22^, ^23^ Participants in the current study had a range of aetiologies, similar to those in trials demonstrating the clinical efficacy of pulmonary rehabilitation in those with bronchiectasis, irrespective of the underlying cause.^19^, ^20^, ^21^ It is acknowledged that disease trajectory in bronchiectasis is variable, that reasons for this disparity are poorly understood and a clearer picture of the natural history and mortality in bronchiectasis is of value.^24^ Requests from people with bronchiectasis to gain greater knowledge and guidance about their disease has been previously expressed.^13^ While it may be assumed by pulmonary rehabilitation clinicians that participants are already informed, our study's findings highlight that patients desire further knowledge on this topic. As the course of disease is dependent on the individual, the role of pulmonary rehabilitation clinicians may be to acknowledge the variability in condition presentation and forecast and emphasise treatment strategies that influence prognosis, which may be adopted.

Discussion and guidance related to self‐management of their condition were also identified as a desired topic for inclusion. This is supported by patient‐centred research priorities from an international registry of bronchiectasis^23^ and recommendations that support and encourage patient empowerment to achieve optimal well‐being and care for their present and future health.^25^ Short‐term improvements in self‐efficacy have been noted in those with bronchiectasis engaging with a self‐management programme.^26^ Those with bronchiectasis have been described as receptive to self‐management, but patient‐reported obstacles include lack of information and resources, particularly in comparison to other disease groups^12^ and low confidence.^13^ A current suggestion from patients is for the provision of self‐management programmes and care plans which enable individuals to have greater control over their condition and recognise and manage exacerbations.^23^ For those individuals with bronchiectasis enrolled in pulmonary rehabilitation, the inclusion of education in self‐management in this setting, delivered by trusted clinicians is a further opportunity to enhance patient understanding and highlight access to resources and possible programme options on this topic, a noted barrier by healthcare professionals regarding the success of self‐management.^27^

Most individuals with bronchiectasis expressed satisfaction regarding their knowledge of airway clearance therapy but identified that the provision of updated information and alternative or adjunct options for airway clearance techniques was desirable. This aligns with research priorities related to improving the awareness and accessibility of airway clearance therapy, optimising instructions in techniques and use of equipment independently.^23^ Despite recommendations for regular engagement with airway clearance therapy,^1^, ^2^, ^3^ adherence to this intervention is low.^28^ While there may be multiple factors influencing adherence, predictors of compliance to airway clearance are linked to beliefs about treatment necessity.^15^ In addition, prescription of airway clearance therapy is complex and no specific technique has been proven superior to another.^29^ A re‐education programme focusing on airway clearance therapy has been previously instituted in people with cystic fibrosis and incorporated treatment rationale, correction of technique, guidance for overcoming barriers and alternative options for airway clearance therapy.^30^ In a modified format, the education programme within pulmonary rehabilitation may provide a further opportunity to reinforce these key concepts and discuss additional or alternative airway clearance therapy options for those with bronchiectasis, aligning with recommendations for the management structure of pulmonary rehabilitation for people with bronchiectasis.^8^ Physical activity levels in those with bronchiectasis are lower compared to healthy peers.^31^ With the higher risk of hospitalisation amongst those with lower physical activity levels,^32^ an opportunity to reiterate the importance of physical activity and engagement with exercise on an ongoing basis is valuable. While this is a common topic included within pulmonary rehabilitation education programmes,^10^ the referral rates to pulmonary rehabilitation of participants in this study were low. This highlights that sourcing additional avenues within the healthcare setting beyond pulmonary rehabilitation may be necessary to maximise understanding of the role and incorporation of regular physical activity for this population.

The psychological burden of bronchiectasis, with anxiety and depression has been previously noted.^33^ Individuals have also illustrated social embarrassment, particularly in relation to symptoms, fear, reduced confidence and poor self‐image.^13^ With these features contributing to a poorer quality of life, it is unsurprising that strategies to assist in managing these features were reported. While coping with a chronic illness has been included within some pulmonary rehabilitation programmes, the psychological impact of lung disease has been reported to be poorly covered in others.^34^ With pulmonary rehabilitation programmes often having a finite number of sessions, it is likely that inherent prioritisation of topics is present and some topics are excluded. To date, it is unclear whether these topics incorporate management options to address psychological symptoms, together with self‐image and coping with symptoms for those with bronchiectasis. Suggestions from the participants in the current study imply greater focus and inclusion of these topics within the education curriculum would be advantageous.

There appears to be an inherent trust in health professionals to deliver relevant educational advice and material,^13^ with greater confidence in receiving information from experienced clinicians.^12^, ^35^ Participants with bronchiectasis also valued educators with sound communication skills, including the ability to target the health literacy of the audience and provide access to supplementary written information, to enhance learning. Each of these strategies has been previously identified as useful by patients with COPD.^11^ However, suggestions for model delivery are mixed; the preference for group sessions aligns with previous reports that group discussion is valued as a method of sharing experiences and receiving peer support^35^ while others prefer individual sessions to maximise relevance, anonymity and privacy.^17^ While group sessions are frequently selected from a staffing perspective, identifying patient preference prior to commencement of pulmonary rehabilitation and determining the ability to accommodate this request, including flexibility in topic delivery, where practical, may require consideration.

Despite the recognition that pulmonary rehabilitation is an evidence‐based treatment for those with bronchiectasis and impaired exercise tolerance,^4^ only a small proportion of patients are offered access to programmes within Australia.^36^ Greater access to these programmes may enhance the opportunity for people with bronchiectasis to engage in education sessions and potentially gain a greater understanding of their disease, the management options and achieve improved self‐efficacy.

This is the first study to examine the educational topics of interest for people with bronchiectasis, within the context of a multifaceted comprehensive pulmonary rehabilitation programme. While a range of ages were included, there was a predominance of females with moderate to severe disease and few had previous exposure to pulmonary rehabilitation. For this reason, the findings presented may not fully reflect the overall disease spectrum of those with bronchiectasis, including those who have been involved in a pulmonary rehabilitation programme. The modified Medical Research Council dyspnoea scores ranged from 0 to 2 in the current study. While some studies included people with bronchiectasis in pulmonary rehabilitation with a modified Medical Research Council dyspnoea score of greater than or equal to 1,^19^, ^20^ current guidelines for pulmonary rehabilitation advocate for inclusion of those with mild respiratory disease (albeit for COPD) and modified Medical Research Council dyspnoea scores less than 1.^4^ In addition, this dyspnoea score is not used as a sole guide for referral to pulmonary rehabilitation for any chronic respiratory condition. It is most common for people with bronchiectasis participating in pulmonary rehabilitation to have comorbidities, including other respiratory conditions, depression and anxiety, all of which are amenable to pulmonary rehabilitation.^4^, ^5^, ^37^ Participants in this study were interviewed prior to the COVID‐19 pandemic; it is possible that additional topics including strategies to avoid infection and alternative models for providing education based on adaptions to pulmonary rehabilitation programmes may also be relevant. Further study is required to establish the patient perspective on these topics in the context of pulmonary rehabilitation programme.

In conclusion, people with bronchiectasis are keen for more information on disease trajectory, self‐management strategies, updates on physiotherapy focused interventions and treatment options related to the psychological burden and symptom stigma of the condition. These unmet education needs provide a foundation for the development of education topics and material which could be offered within a pulmonary rehabilitation setting.

## CONFLICT OF INTEREST

All authors have no disclosures or conflicts of interest to declare.

## ETHICS STATEMENT

This study has been approved by the institutional review board at Monash University (approval HREC 18416). All participating patients were requested to provide written informed consent.

## AUTHOR CONTRIBUTIONS

ALL conceived the study design, undertook the methodology, conducted the formal analysis and drafted the manuscript. RS and RMcA conceived the study, undertook formal analysis and reviewed and edited the manuscript. LB, ABC, C‐L H‐L, PK, PM, LM, DS, RT, GW and CW reviewed and edited the manuscript. All authors reviewed and approved the final manuscript to be published.

## Data Availability

The data supporting the findings of this study are available on reasonable request from the corresponding author. The data are not publicly available due to privacy or ethical restrictions.

## APPENDIX A INTERVIEW QUESTIONS

**Table:**

Interview questions
How would you describe your understanding of your lung condition? What do you think you would like to learn about your condition in the future? What kind of information do you think it is really essential for people with bronchiectasis to know about their condition? Some people receive information about bronchiectasis when they attend a pulmonary rehabilitation programme. Have you ever attended a pulmonary rehabilitation programme? If yes, can you describe the information you received about bronchiectasis in pulmonary rehabilitation? If no, can you tell me what you understand or know about pulmonary rehabilitation? What kind of information do you think it would be useful to cover during pulmonary rehabilitation? Education sessions in pulmonary rehabilitation are conducted in a group of people with a range of different chronic lung conditions. What do you think about this format for education sessions? Do you have a preference for group or individual sessions? Can you elaborate on the reason for your choice? Do you have any thoughts on which health professionals should provide education in pulmonary rehabilitation? Do you have any suggestions as to how best to retain the knowledge learnt during the education sessions from your perspective? If you could give health professionals one piece of advice about educating people with bronchiectasis about their condition, what would it be?
